# Novel Prognostic Score for recurrent or metastatic head and neck cancer patients treated with Nivolumab

**DOI:** 10.1038/s41598-021-96538-7

**Published:** 2021-08-20

**Authors:** Kiyoshi Minohara, Takuma Matoba, Daisuke Kawakita, Gaku Takano, Keisuke Oguri, Akihiro Murashima, Kazuhiro Nakai, Sho Iwaki, Wataru Hojo, Ayano Matsumura, Shinya Ozaki, Taijiro Ozawa, Ikuma Harata, Nobukazu Tanaka, Shinichiro Maseki, Hiroshi Tsuge, Sae Imaizumi, Shoji Mitsuya, Kazuho Moribe, Shinichi Esaki, Shinichi Iwasaki

**Affiliations:** 1 Department of Otorhinolaryngology, Head and Neck Surgery, Nagoya City University Graduate School of Medical Sciences, 1 Kawasumi, Mizuho-cho, Mizuho-ku, Nagoya, Aichi 467-8601 Japan; 2 Department of Otorhinolaryngology, Konan Kosei Hospital, Konan, Japan; 3 Department of Otorhinolaryngology, Toyohashi Municipal Hospital, Toyohashi, Japan; 4 Department of Otorhinolaryngology, Kainan Hospital, Yatomi, Japan; 5 Department of Otorhinolaryngology, Toyota Kosei Hospital, Toyota, Japan; 6 Department of Otorhinolaryngology, Anjo Kosei Hospital, Anjo, Japan; 7 Department of Otorhinolaryngology, Ichinomiya Municipal Hospital, Ichinomiya, Japan

**Keywords:** Chemotherapy, Prognostic markers, Head and neck cancer, Immunotherapy, Cancer

## Abstract

Although several prognostic factors in nivolumab therapy have been reported in recurrent or metastatic head and neck cancer (RM-HNC) patients, these factors remain controversial. Here, we conducted a multicenter retrospective cohort study to investigate the impact of clinico-hematological factors on survival in RM-HNC patients treated with nivolumab. We reviewed 126 RM-HNC patients from seven institutes. We evaluated the prognostic effects of clinico-hematological factors on survival. The median overall survival (OS) was 12.3 months, and the 1 year-OS rate was 51.2%. Patients without immune-related adverse events, lower relative eosinophil count, worse best overall response, higher performance status, and higher modified Glasgow Prognostic Score had worse survival. The score, generated by combining these factors, was associated with survival. Patients with score of 4–5 had worse survival than those with score of 2–3 and 0–1 [adjusted HR for PFS: score of 4–5, 7.77 (3.98–15.15); score of 2–3, 3.44 (1.95–6.06), compared to score of 0–1], [adjusted HR for OS: score of 4–5, 14.66 (4.28–50.22); score of 2–3, 7.63 (2.29–25.37), compared to score of 0–1]. Our novel prognostic score utilizing clinico-hematological factors might be useful to establish an individual treatment strategy in RM-HNC patients treated with nivolumab therapy.

## Introduction

Immune checkpoint inhibitors (ICIs) have become standard therapy for various types of cancer^1–4^. Nivolumab, a programmed cell death 1 (PD-1) inhibitor, was approved in 2017 to treat platinum-refractory recurrent or metastatic head and neck cancer (RM-HNC) in Japan, receiving the results of CheckMate 141 trial^1^. Although nivolumab is highly effective in some patients, more than half of these patients do not have clinical benefits.

To date, several studies have reported the association of patient-related factors, including the best overall response (BOR), the occurrence of immune-related adverse events (irAEs), programmed death-ligand 1 (PD-L1) expression, high mutational burden, hematological inflammatory, and nutritional markers, and clinical outcomes in RM-HNC patients treated with nivolumab therapy^5–13^. Although several studies reported the utility of the combination of these factors in ICIs therapy, we found only one study in head and neck caner^13–17^. Therefore, the optimal prognostic factors for nivolumab therapy in patients with RM-HNC are still controversial. Furthermore, in considering the cost-effectiveness of ICI therapy, it is essential to elucidate the optimal prognostic factors in RM-HNC patients treated with ICI therapy.

Here, we conducted a multicenter retrospective cohort study to investigate the impact of clinico-hematological factors on survival in RM-HNC patients treated with nivolumab therapy among the Japanese population.

## Results

### Patient characteristics

The median follow-up interval was 7.5 months (range 0.5–33 months) for all patients in this study. The patient characteristics are summarized in Table 1. The median age was 68 years (range 35–90 years), and men were predominant. The primary tumor sites were oral cavity cancer in 12, oropharyngeal cancer in 27, hypopharyngeal cancer in 20, laryngeal cancer in 21, sinonasal cancer in 13, nasopharyngeal cancer in 11, salivary gland cancer in 8, external ear canal cancer in 7, and unknown primary cancer in 7. Fifty-four patients had locoregional diseases, and 72 patients had distant metastases. Regarding Eastern Cooperative Oncology Group Performance Status (ECOG PS), most patients were less than 1.

**Table 1:** Patient characteristics with nivolumab in recurrent or metastatic head and neck cancer.

Variables	N (= 126)	%
**Age**		
< 69	67	53
≥ 69	59	47
**Sex**		
Male	104	83
Female	22	17
**Primary tumor site**		
Oral cavity	12	9
Nasopharynx	11	9
Oropharynx	27	21
Hypopharynx	20	16
Larynx	21	17
Sinonasal cavity	13	10
Salivary gland	8	6
External ear canal	7	6
Unknown	7	6
**ECOG PS**		
01	102	81
2–3	24	19
**Site of recurrence**		
Loco-regional	54	43
Distant	72	57
**Platinum sensitivity**		
Sensitive	58	46
Refractory	68	54
**Modified Glasgow Prognostic Score**		
0	70	56
1	12	10
2	35	27
Unknown	9	7
**Relative eosinophil count**		
< 1.5	71	56
≥ 1.5	53	42
Unknown	2	2
**Neutrophil/lymphocyte ratio**		
< 5	71	56
≥ 5	53	43
Unknown	1	1
**Platelet/lymphocyte ratio**		
< 253	62	49
≥ 253	63	50
Unknown	1	1

ECOG PS, Eastern Cooperative Oncology Group Performance Status.

### IrAE profile

The irAE profiles are shown in Supplementary Table S1. Forty-one patients (32.5%) presented with 50 irAEs. The most common irAE category was endocrine irAEs (hypothyroidism, hypophysitis, etc.), followed by skin irAEs (rash, dermatitis, rash acneiform, etc.). There were 24 grade 3 or higher irAEs, and steroid therapy was administered to 25 patients.

### Clinical outcomes

For all the patients, the median overall survival (OS) was 12.3 months (95% CI 9.7–16.0), and 1 year-OS rate was 51.2% (95% CI 40.0–61.2), and the median progression-free survival (PFS) was 3.9 months (95% CI 2.8–5.4) and 1 year-PFS rate was 14.6% (95% CI 8.7–22.0) (Supplementary Fig. S1). BOR to nivolumab therapy was complete response (CR) in 5 patients (4.0%), partial response (PR) in 24 patients (19.0%), stable disease (SD) in 48 patients (38.1%), and progressive disease (PD) in 49 patients (38.9%). After PD in nivolumab therapy, chemotherapy was administered to 44 patients. Among these patients, paclitaxel and cetuximab therapy was the most common (47.7%) (Supplementary Table S2). BOR to chemotherapy after nivolumab was CR in 1 patient (2.3%), PR in 17 patients (38.6%), SD in 6 patients (13.6%), and PD in 19 patients (43.2%).

The association between clinico-hematological factors and clinical outcomes is shown in Table 2. In univariate analysis, OS was better for patients with any irAEs (HR 0.49; 95%CI, 0.27–0.87; *p* = 0.016), higher REC (HR 0.51, 95% CI 0.31–0.86, *p* = 0.011), and better BOR (HR 0.18, 95% CI 0.08–0.41, *p* < 0.001). Moreover, OS was worse in patients with higher ECOG PS (HR 3.14; 95%CI, 1.77–5.57; *p* < 0.001) and higher modified Glasgow Prognostic Score (mGPS) (HR 2.40, 95% CI 1.41–4.10, *p* = 0.001). Furthermore, PFS was better for patients with any irAEs (HR 0.55, 95% CI 0.36–0.84, *p* = 0.006), higher relative eosinophil count (REC) (HR 0.68, 95% CI 0.46–1.00, *p* = 0.049), and better BOR (HR 0.19, 95% CI 0.11–0.33, *p* < 0.001). Additionally, PFS was worse for patients with higher ECOG PS (HR 1.90; 95%CI, 1.18–3.06; *p* = 0.008) and higher mGPS (HR 2.02, 95% CI 1.34–3.03, *p* < 0.001).

**Table 2:** Impact of clinical, hematological factors and prognostic score on clinical outcomes in recurrence or metastatic head and neck cancer patients treated with nivolumab.

Variables	N	Progression-free survival						Overall survival					
		Univariate			Multivariate			Univariate			Multivariate		
		HR	95% CI	*p*-value	HR	95% CI	*p*-value	HR	95% CI	*p*-value	HR	95% CI	*p*-value
**ECOG PS**													
0, 1	102	1.00	–	–	1.00	–	–	1.00	–	–	1.00	–	–
2, 3	24	1.90	1.18–3.06	0.008	2.17	1.30–3.63	0.003	3.14	1.77–5.57	< 0.001	2.94	1.55–5.55	< 0.001
**IrAE**													
Without	85	1.00	–	–	1.00	–	–	1.00	–	–	1.00	–	–
With	41	0.55	0.36–0.84	0.006	0.52	0.34–0.80	0.003	0.49	0.27–0.87	0.016	0.47	0.26–0.86	0.015
**Modified Glasgow Prognostic Score**													
0	70	1.00	–	–	1.00	–	–	1.00	–	–	1.00	–	–
1, 2	47	2.02	1.34–3.03	< 0.001	2.37	1.55–3.63	< 0.001	2.40	1.41–4.10	0.001	2.51	1.45–4.35	< 0.001
**Relative eosinophil count**													
< 1.5	71	1.00	–	–	1.00	–	–	1.00	–	–	1.00	–	–
≥ 1.5	53	0.68	0.46–1.00	0.049	0.68	0.46–1.00	0.048	0.51	0.31–0.86	0.011	0.49	0.29–0.83	0.008
**Neutrophil/lymphocyte ratio**													
< 5	71	1.00	–	–	1.00	–	–	1.00	–	–	1.00	–	–
≥ 5	53	1.28	0.87–1.88	0.218	1.32	0.89–1.95	0.167	1.54	0.93–2.56	0.094	1.56	0.92–2.65	0.096
**Platelet/lymphocyte ratio**													
< 253	62	1.00	–	–	1.00	–	–	1.00	–	–	1.00	–	–
≧ 253	63	1.04	0.71–1.53	0.830	1.10	0.75–1.63	0.626	1.37	0.82–2.29	0.236	1.37	0.80–2.35	0.246
**Best overall response**													
SD, PD	97	1.00	–	–	1.00	–	–	1.00	–	–	1.00	–	–
CR, PR	29	0.19	0.11–0.33	< 0.001	0.17	0.10–0.30	< 0.001	0.18	0.08–0.41	< 0.001	0.18	0.07–0.42	< 0.001
**Prognostic score**													
0–1	27	1.00	–	–	1.00	–	–	1.00	–	–	1.00	–	–
2–3	61	2.78	1.60–4.83	< 0.001	3.44	1.95–6.06	< 0.001	6.67	2.04–21.90	< 0.001	7.63	2.29–25.37	< 0.001
4–5	28	6.11	3.22–11.61	< 0.001	7.77	3.98–15.15	< 0.001	15.06	4.43–51.13	< 0.001	14.66	4.28–50.22	< 0.001
		P_trend_ < 0.001			P_trend_ < 0.001			P_trend_ < 0.001			P_trend_ < 0.001		

Adjusted for age, sex, primary tumor site, platinum sensitivity, and site of recurrence.

*ECOG PS* Eastern Cooperative Oncology Group Performance Status, *irAE* immune-related adverse events.

In multivariate analysis, OS was better for patients with any irAEs (HR 0.47, 95% CI 0.26–0.86, *p* = 0.015), higher REC (HR 0.49, 95% CI 0.29–0.83, *p* = 0.008), and better BOR (HR 0.18, 95% CI 0.07–0.42, *p* < 0.001). Besides, OS was worse in patients with higher ECOG PS (HR 2.94; 95%CI, 1.55–5.55; *p* < 0.001) and higher mGPS (HR 2.51, 95% CI 1.45–4.35, *p* < 0.001). Furthermore, PFS was better for patients with any irAEs (HR 0.52, 95% CI 0.34–0.80, *p* = 0.003), higher REC (HR 0.68, 95% CI 0.46–1.00, *p* = 0.048), and better BOR (HR 0.17, 95% CI 0.10–0.30, *p* < 0.001). Additionally, PFS was worse for patients with higher ECOG PS (HR 2.17; 95% CI 1.30–3.63, *p* = 0.003) and higher mGPS (HR 2.37, 95% CI 1.55–3.63, *p* < 0.001).

### The impact of prognostic score using the sum of numbers from clinico-hematological factors

**Figure 1:**
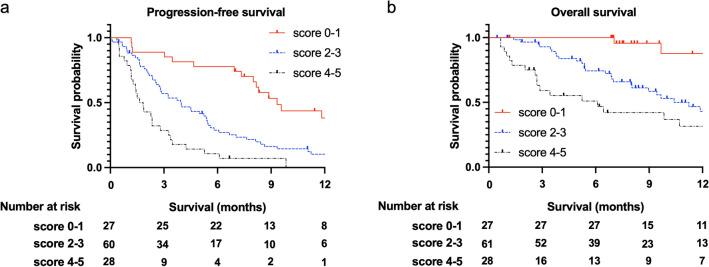
Kaplan–Meier curves of progression-free survival (PFS) and overall survival (OS) in patients with recurrent or metastatic head and neck cancer treated with nivolumab, stratified by prognostic score. Prognostic score is the sum of numbers from worse prognostic factors in clinico-hematological factors including without immune-related adverse events (irAEs), lower relative eosinophil count (REC), worse best overall response (BOR), higher Eastern Cooperative Oncology Group Performance Status (ECOG PS), and higher modified Glasgow Prognostic Score (mGPS). (**a**) Patients with a prognostic score of 4–5 (N = 28) had significantly worse PFS than those with prognostic scores of 2–3 (N = 61) and 0–1 (N = 27) (1 year-PFS: 0.0% vs. 10.1% [95% CI 3.9–19.8] vs. 38.2% [95% CI 18.9–57.4], *p* < 0.001). (**b**), Patients with a prognostic score of 4–5 (N = 28) had significantly worse OS than those with prognostic scores of 2–3 (N = 61) and 0–1 (N = 27) (1 year-OS: 26.3% [95% CI 10.3–45.6] vs. 43.1% [95% CI 27.4–57.9] vs. 87.7% [95% CI 56.9–97.0], *p* < 0.001).

Finally, we investigated the impact of prognostic score using the sum of numbers from worse prognostic factors that were statistically significant in clinico-hematological factors, including without irAEs, lower REC, worse BOR, higher ECOG PS, and higher mGPS (Fig. 1 and Table 2). We found that higher scores were associated with worse OS and PFS, with a significant trend (*p* for trend < 0.001). Patients with score of 4–5 had worse survival than those with score of 2–3 and 0–1 [1 year-PFS: 0.0% vs. 10.1% vs. 38.2%, respectively; adjusted HR score of 4–5, 7.77 (95% CI 3.98–15.15); score of 2–3, 3.44 (95% CI 1.95–6.06), compared to score of 0–1], [1 year-OS: 26.3% vs. 43.1% vs. 87.7%, respectively; adjusted HR score of 4–5, 14.66 (95% CI 4.28–50.22); score of 2–3, 7.63 (95% CI 2.29–25.37), compared to score of 0–1].

## Discussion

This study found the prognostic effect of clinico-hematological factors, including the occurrence of irAEs, REC, BOR, ECOG PS, and mGPS in RM-HNC patients treated with nivolumab therapy. Furthermore, our analysis showed that the sum of numbers from worse prognostic factors in clinico-hematological factors might be the optimal prognostic score in the Japanese population.

Similar to previous studies reported^5,18^, we found that the occurrence of irAEs was significantly associated with better prognosis in RM-HNC patients treated with nivolumab therapy. Matsuo et al. reported that gastrointestinal irAEs were significantly associated with better PFS^5^. In other types of cancer, cutaneous, gastrointestinal, and endocrine irAEs are associated with better survival^19,20^. Although the endocrine irAEs category was most common in this study, we could not detect which type of irAE was strongly associated with clinical outcomes. However, since appropriate management of irAEs could lead to the clinical benefit of nivolumab therapy, early detection of irAEs and management in multidisciplinary teams should be advocated.

Additionally, we found that a better BOR to nivolumab therapy was associated with better survival. In the CheckMate 141 trial, the response rate in the nivolumab group was higher than that in the investigator's choice group, and tumor reduction was more durable with nivolumab^1^. Matsuki et al. also demonstrated that a better BOR was significantly associated with better survival^21^. It is obvious that response evaluation is important even when using immunotherapeutic agents.

Consistent with our results, Nishikawa et al. demonstrated that higher eosinophil counts and increases were associated with better survival^13,22^. Furthermore, eosinophil accumulation was associated with better survival in patients with melanoma treated with ICI^23^. They mentioned that the high number of peripheral blood eosinophils might reflect the high number of tumor-infiltrating eosinophils and the increase in tumor antigens due to tumor necrosis and collapse.

In this study, modified GPS and ECOG PS were associated with clinical outcomes in RM-HNC patients treated with nivolumab therapy. These factors are known to be prognostic factors in patients with ICI and other treatments^24,25^. Therefore, these biomarkers may be useful in various treatment modalities. The advantage of these biomarkers and eosinophil count, described above, is that they can be evaluated before nivolumab treatment. Since these two factors indicate the patient's general condition and/or inflammation, it might be better to use ICI without any symptoms associated with RM-HNC.

Some studies have also reported the utility of the combination of each factor in several type of cancer treated with ICIs. In non–small cell lung cancer patients treated with anti-PD-1/PD-L1 antibodies, the ESPILoN score [smoking history, liver metastasis, lactate dehydrogenase (LDH), and neutrophil to lymphocyte ratio (NLR)], the Gustave Roussy Immune Score (GRIm-Score) (LDH, serum albumin (Alb), and NLR) and the RHM score (LDH, Alb, and the number of metastatic sites) was reported to be significantly correlated with prognosis^14–16^. The Emory risk scoring system including the monocyte-to-lymphocyte ratio (MLR), body mass index (BMI) and number of metastatic sites was significantly associated with survival in recurrent or metastatic renal cell carcinoma treated with anti-PD-1 antibodies^17^. In recurrent or metastatic head and neck squamous cell carcinoma treated with anti-PD-1 antibodies, the Eosinophil Prognostic Score (REC, the ratio of eosinophil increase, and ECOG PS) was reported to be significantly correlated with survival^13^. We demonstrated that the sum of numbers from worse prognostic factors could be the optimal prognostic score in RM-HNC patients treated with nivolumab. To the best of our knowledge, this is the first report to evaluate the impact of prognostic score associated with clinico-hematological factors, which are routinely available in clinical settings and included irAE and BOR, on survival in RM-HNC patients with nivolumab. However, the concern is that the poor nutritional condition and infection could affect these factors. Therefore, supportive therapy, including nutritional support, oral care, and smoking cessation, should be considered in patients with RM-HNC.

Regarding the primary tumor site, while we performed nivolumab therapy in patients not included in the CheckMate 141 trial, such as nasopharyngeal cancer, clinical outcomes were comparable to those included in the CheckMate 141 trial^1,7^. In patients excluded from the CheckMate 141 trial, the efficacy of nivolumab therapy has been reported with primary tumors at other sites, including the nasopharynx, while its efficacy is limited in the salivary gland cancer^5,26–28^. However, there is insufficient evidence for the optimal systematic therapy for HNC other than squamous cell carcinoma (SCC). A larger collaborative study to evaluate the efficacy of nivolumab therapy in patients with HNC other than SCC is required.

Regarding salvage chemotherapy following nivolumab therapy, we mainly performed paclitaxel and cetuximab therapy, and our clinical outcomes, including 41% ORR, were comparable to those of previous studies^29–31^. As mentioned above, since the efficacy of cetuximab and other chemotherapies following ICI has been reported, relatively early changes in treatment modalities might be acceptable.

Our study had several strengths. Since this is a multi-institutional cohort study, our sample size is one of the largest studies in patients with RM-HNC in a real-world setting. Second, detailed individual data were available for this study. Third, because physicians had no information on the association of clinic-hematological factors with survival, information bias appears unlikely. Fourth, we could perform the analysis adjusted for potential confounders, including detailed clinical information.

Moreover, several limitations should be mentioned. First, this study was conducted with a retrospective and multi-institutional design. Second, our information, especially hematological factors, reflected pre-treatment status only and not post-treatment factors, which might be associated with clinical outcomes. Third, we could not fully remove the potential effect of factors of infectious diseases, inflammation other than that derived from RM-HNC, and the use of glucocorticoid hormones. To overcome these limitations, future large scale, multicenter and prospective studies are warranted.

In conclusion, we found that the occurrence of irAEs, higher REC, better BOR, lower ECOG PS, and lower mGPS were the better prognostic factors for survival in patients with RM-HNC treated with nivolumab. Furthermore, the sum of the numbers of worse prognostic factors might be the optimal prognostic score. Using this novel prognostic score, more effective treatment strategies, including nivolumab therapy, could be established for patients with RM-HNC.

## Materials and methods

### Patients

We conducted a retrospective cohort study to investigate the efficacy and safety of nivolumab therapy in RM-HNC patients in seven institutes, including Nagoya City University Hospital, Konan Kosei Hospital, Toyohashi Municipal Hospital, Kainan Hospital, Toyota Kosei Hospital, Anjo Kosei Hospital, and Ichinomiya Municipal Hospital. Among these institutes, 126 RM-HNC patients were treated with nivolumab between April 2017 and November 2019. The total of 10 patients who lacked information on clinico-hematological factors were excluded. Finally, 116 patients were enrolled in the prognostic score analysis. The Institutional Review Board (IRB) of Nagoya City University Graduate School of Medical Sciences approved our protocols (approval number: 60-21-0001). Concerning consent to participate, patients could reject participation by opting out, to an announcement on Nagoya City University Hospital’s website. This study was conducted in accordance with the principles of the Declaration of Helsinki.

### Treatment and follow-up

All patients had been treated with platinum-based therapy before nivolumab therapy. Nivolumab was administered at a dose of 3 mg/kg every 2 weeks. The response to nivolumab therapy was evaluated according to Response Evaluation Criteria in Solid Tumor (RECIST) criteria version 1.1^32^, using computed tomography (CT) or magnetic resonance imaging (MRI) every 8–12 weeks. Patients in whom nivolumab administration was terminated due to clinically obvious disease progression were diagnosed with PD, even when image evaluation was not performed. Chemotherapy with cytotoxic agents was administered to patients diagnosed with PD. Follow-up was continued until death or the cut-off date (May 6, 2020).

### Prognostic effect of clinical and hematological factors

This study evaluated the prognostic effect of clinical factors, including age, sex, primary tumor site, ECOG PS, site of recurrence, and platinum sensitivity. A platinum-refractory tumor was defined as a tumor that progressed within 6 months after the last platinum-based chemotherapy or a residual tumor after platinum-based chemoradiotherapy. A platinum-sensitive tumor was defined as a tumor that progressed from 6 months or longer after the last platinum-based chemotherapy. Moreover, we also evaluated the prognostic effect of hematological factors: REC, NLR, PLR, and mGPS. Hematological factors were calculated by eosinophil count, neutrophil count, lymphocyte count, platelet count, C-reactive protein (CRP), and Alb in peripheral blood just before the start of nivolumab therapy. Regarding mGPS, patients with both elevated CRP level (> 1.0 mg/dL) and decreased Alb (< 3.5 g/dL) were assigned a score of 2; those with elevated CRP level (> 1.0 mg/dL) and non-decreased Alb (≥ 3.5 g/dL) were assigned a score of 1, and those with a non-elevated CRP level (≤ 1.0 mg/dL) were assigned a score of 0 according to a previous study^33^.

### Statistical analysis

We investigated overall effectiveness, including BOR, PFS, and OS. BOR was defined as the best response from the initiation of nivolumab administration to PD. PFS was defined as the time from the first nivolumab administration to the date of PD or clinically unequivocal progression. OS was defined as the time from the first nivolumab administration to the date of death or the last visit. The irAEs were evaluated according to the protocol described in a previous study^34^. Toxicity was assessed using the Common Terminology Criteria for Adverse Events (CTCAE) version 5.0^35^.

Additionally, we evaluated the frequency of irAEs and the association between clinico-hematological factors and clinical outcomes in nivolumab therapy. The association of clinico-hematological factors with PFS or OS was assessed using the Kaplan–Meier product-limit method and univariate and multivariate Cox proportional hazards models. In the multivariate analysis, the forced entry method was performed. The measure of association in this study was hazard ratios (HRs) with 95% confidence intervals (CIs). Statistical significance was set at *P* < 0.05. Statistical analyses were performed using GraphPad Prism (version 9.00; GraphPad Software, San Diego, CA, USA) and EZR version 1.40 (Saitama Medical Center, Jichi Medical University, Saitama, Japan), which is a graphical user interface for R (R version 3.6.1, The R Foundation for Statistical Computing, Vienna, Austria)^36^. More precisely, it is a modified version of R commander designed to add statistical functions frequently used in biostatistics.

### Ethical approval

The study protocol was approved by the Institutional Review Board (IRB) of Nagoya City University Graduate School of Medical Sciences (# 60-21-0001). Concerning consent to participate, patients could reject participation by opting out, to an announcement on Nagoya City University Hospital’s website. Therefore, written informed consent was waived, which was approved by the IRB of Nagoya City University Graduate School of Medical Sciences. This study was conducted in accordance with the principles of the Declaration of Helsinki.

## Supplementary Information

Supplementary Information.

## Data Availability

The datasets generated in this study are available from the corresponding author on request.
