# Supplementary material for: Novel Prognostic Score for recurrent or metastatic head and neck cancer patients treated with Nivolumab
Source: Sci Rep. 2021 Aug 20;11:16992. doi: 10.1038/s41598-021-96538-7 (PMC8379150; doi:10.1038/s41598-021-96538-7)
Supplement: Supplementary file 1 — Supplementary Information. [file 41598_2021_96538_MOESM1_ESM.pdf]

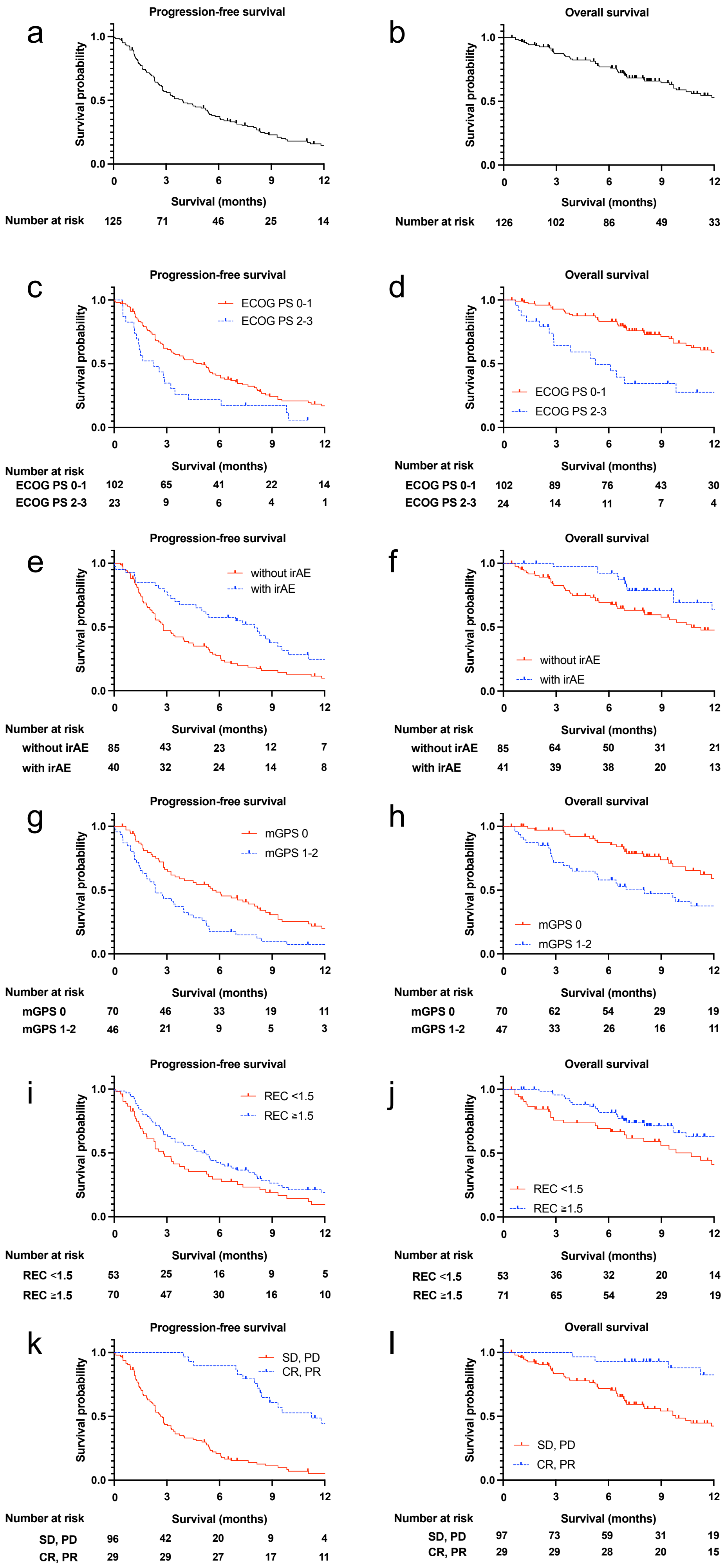
a
Progression-free survival
Survival probability
1.0
0.5
0.0
0
3
6
9
12
Survival (months)
Number at risk 125 71 46 25 14
b
Overall survival
Survival probability
Survival (months)
Number at risk 126 102 86 49 33
c
Progression-free survival
ECOG PS 0-1
ECOG PS 2-3
Survival probability
Survival (months)
Number at risk
ECOG PS 0-1 102 65 41 22 14
ECOG PS 2-3 23 9 6 4 1
d
Overall survival
ECOG PS 0-1
ECOG PS 2-3
Survival probability
Survival (months)
Number at risk
ECOG PS 0-1 102 89 76 43 30
ECOG PS 2-3 24 14 11 7 4
e
Progression-free survival
without irAE
with irAE
Survival probability
Survival (months)
Number at risk
without irAE 85 43 23 12 7
with irAE 40 32 24 14 8
f
Overall survival
without irAE
with irAE
Survival probability
Survival (months)
Number at risk
without irAE 85 64 50 31 21
with irAE 41 39 38 20 13
g
Progression-free survival
mGPS 0
mGPS 1-2
Survival probability
Survival (months)
Number at risk
mGPS 0 70 46 33 19 11
mGPS 1-2 46 21 9 5 3
h
Overall survival
mGPS 0
mGPS 1-2
Survival probability
Survival (months)
Number at risk
mGPS 0 70 62 54 29 19
mGPS 1-2 47 33 26 16 11
i
Progression-free survival
REC <1.5
REC ≧1.5
Survival probability
Survival (months)
Number at risk
REC <1.5 53 25 16 9 5
REC ≧1.5 70 47 30 16 10
j
Overall survival
REC <1.5
REC ≧1.5
Survival probability
Survival (months)
Number at risk
REC <1.5 53 36 32 20 14
REC ≧1.5 71 65 54 29 19
k
Progression-free survival
SD, PD
CR, PR
Survival probability
Survival (months)
Number at risk
SD, PD 96 42 20 9 4
CR, PR 29 29 27 17 11
l
Overall survival
SD, PD
CR, PR
Survival probability
Survival (months)
Number at risk
SD, PD 97 73 59 31 19
CR, PR 29 29 28 20 15

Supplementary Figure S1.

Kaplan–Meier curves of progression-free survival (a, c, e, g, i, k) and overall survival (b, d, f, h, j, l) in recurrence or metastatic head and neck cancer patients treated with nivolumab. Kaplan–Meier curves of progression-free survival and overall survival in (a, b) all patients, stratified by (c, d) Eastern Cooperative Oncology Group Performance Status (ECOG PS), (e, f) immune-related adverse events (irAE), (g, h) modified Glasgow prognostic score (mGPS), (i, j) Relative Eosinophil Count (REC), (k, l) best overall response (BOR). The vertical lines indicate censored events. Among all patients (N=126), (a) 1 year-PFS rate was 14.6% (95%CI: 8.7-22.0), and (b) 1 year-OS rate was 51.2% (95%CI: 40.0-61.2). (c), Patients with high ECOG PS 2-3 (N=24) had significantly worse PFS than ECOG PS 0-1 (N=102) (1 year-PFS: 0.0% vs 17.0% (95% CI, 10.0-25.5), p= 0.007). (d), Patients with high ECOG PS 2-3 (N=24) had significantly worse PFS than ECOG PS 0-1 (N=102) (1 year-OS: 27.6% (95% CI, 10.2-48.4) vs 56.5% (95% CI, 43.7-67.5), p< 0.001). (e), Patients without irAE (N=85) had significantly worse OS than with irAE (N=41) (1 year-PFS: 9.9% (95% CI, 4.4-18.0) vs 24.1% (95% CI, 11.6-39.1), p= 0.005). (f), Patients without irAE (N=85) had significantly worse OS than with irAE (N=41) (1 year-OS: 45.2% (95% CI, 32.2-57.4) vs 64.0% (95% CI, 42.7-79.1), p= 0.014). (g), Patients with mGPS 1-2 (N=47) had significantly worse PFS than with mGPS 0 (N=70) (1 year-PFS: 7.3% (95% CI, 2.0-17.5) vs 19.8% (95% CI, 10.8-30.7), p< 0.001). (h), Patients with mGPS 1-2 (N=47) had significantly worse OS than with mGPS 0 (N=70) (1 year-OS: 33.3% (95% CI, 18.4-49.0) vs 59.1% (95% CI, 42.8-72.2), p< 0.001). (i), Patients with REC < 1.5 (N=53) had significantly worse PFS than with REC ≥ 1.5 (N=71) (1 year-PFS: 9.6% (95% CI, 3.2-20.2) vs 18.8% (95% CI, 10.2-29.4), p= 0.047). (j), Patients with REC < 1.5 (N=53) had significantly worse OS than with REC ≥ 1.5 (N=71) (1 year-OS: 37.7% (95% CI, 22.4-53.0) vs 63.1% (95% CI, 48.2-74.8), p= 0.010). (k), Patients with SD, PD (N=97) had significantly worse PFS than with CR, PR(N=29) (1 year-PFS: 5.2% (95% CI, 1.6-12.0) vs 44.3% (95% CI, 25.0-62.0), p< 0.001). (l), Patients with SD, PD (N=97) had significantly worse OS than with CR, PR(N=29) (1 year-OS: 40.0% (95% CI, 27.7-52.0) vs 82.4% (95% CI, 58.7-93.2), p< 0.001).

Supplementary Table S1. IrAE profiles with nivolumab in recurrent or metastatic head and neck cancer.

IrAE (system organ class)	Number of events					
	Grade 1, 2	%	Grade 3, 4	%	Total	%
Skin	7	14	4	8	11	22
Endocrine	7	14	5	10	12	24
Interstitial lung disease	5	10	4	8	9	18
Gastro-intestinal	4	8	3	6	7	14
Infusion reaction	1	2	2	4	3	6
Hepatic	1	2	3	6	4	8
Myasthenia gravis	0	0	1	2	1	2
Others	1	2	2	4	3	6
Total	26	52	24	48	50	100

Abbreviation: irAE, immune-related adverse event.

Supplementary Table S2. The next chemotherapy after nivolumab in recurrence or metastatic head and neck cancer patients.

Rejimen	N (=44)	%
Paclitaxel+Cetuximab	21	47.7
Tegafur/Gimeracil/Oteracil	9	20.5
Paclitaxel	7	15.9
Cisplatin+5-FU+Cetuximab	2	4.5
Cisplatin+5-FU	1	2.3
Cetuximab	1	2.3
Carboplatin+Paclitaxel	1	2.3
Boron Neutron Capture Therapy	1	2.3
Docetaxel	1	2.3
